# Genome-wide identification and development of miniature inverted-repeat transposable elements and intron length polymorphic markers in tea plant (*Camellia sinensis*)

**DOI:** 10.1038/s41598-022-20400-7

**Published:** 2022-09-28

**Authors:** Megha Rohilla, Abhishek Mazumder, Dipnarayan Saha, Tarun Pal, Shbana Begam, Tapan Kumar Mondal

**Affiliations:** 1 ICAR-National Institute for Plant Biotechnology, LBS Centre, Pusa, New Delhi, 110012 India; 2 Division of Crop Improvement, ICAR-Central Research Institute for Jute and Allied Fibres, Barrackpore, Kolkata, West Bengal 700121 India

**Keywords:** Gene delivery, Genomics, Plant biotechnology

## Abstract

Marker-assisted breeding and tagging of important quantitative trait loci for beneficial traits are two important strategies for the genetic improvement of plants. However, the scarcity of diverse and informative genetic markers covering the entire tea genome limits our ability to achieve such goals. In the present study, we used a comparative genomic approach to mine the tea genomes of *Camellia sinensis* var*. assamica* (CSA) and *C. sinensis* var*. sinensis* (CSS) to identify the markers to differentiate tea genotypes. In our study, 43 and 60 *Camellia sinensis* miniature inverted-repeat transposable element (CsMITE) families were identified in these two sequenced tea genomes, with 23,170 and 37,958 putative CsMITE sequences, respectively. In addition, we identified 4912 non-redundant, *Camellia sinensis* intron length polymorphic (CsILP) markers, 85.8% of which were shared by both the CSS and CSA genomes. To validate, a subset of randomly chosen 10 CsMITE markers and 15 CsILP markers were tested and found to be polymorphic among the 36 highly diverse tea genotypes. These genome-wide markers, which were identified for the first time in tea plants, will be a valuable resource for genetic diversity analysis as well as marker-assisted breeding of tea genotypes for quality improvement.

## Introduction

Tea is an important plantation crop in India and is widely consumed as a non-alcoholic beverage around the world. As tea is a perennial, woody, cross-pollinated plant^1^, the conventional breeding program is extremely slow. Being a recalcitrant plant (i.e., difficult to regenerate in vitro), the transgenic or genome-editing approach for genetic improvement of tea is difficult^2^. Modern tea cultivars still rely primarily on hybridization as a method of genetic improvement. There are three botanical subgroups of tea plants (i.e., Assam, China, and Cambod type) based on morphological parameters, but due to their high outcrossing nature, they can all interbreed freely. The existing tea population today is mostly genetic admixtures of these three types^3^. Therefore, estimating the purity of tea genotypes using molecular markers is an important criterion for precious tea breeding.

Tea breeding is restricted to clonal selection of superior bush from the existing natural population. A systematic breeding technique for tea genetic improvement is not obscure. It is noteworthy to mention that a few draft genomes of tea, including the Assam and China types, have been reported^4,5^, providing insights into the tea genome's organization and genetic information. The development of molecular markers using the draft genomes of these two cultivars is one of the useful strategies for tagging the important QTLs and marker-assisted breeding for agronomically important traits. The development of a large number of diverse and informative genetic markers to cover the entire tea genome is thus necessary to accomplish such goals. Several DNA markers in tea plant, such as randomly amplified polymorphic DNA (RAPD), inter simple sequence repeats (ISSR), amplified fragment length polymorphism (AFLP), and simple sequence repeats (SSR), were reported primarily from the pre-genome sequence information^6^. However, they are insufficient to saturate the whole tea genome due to their large genome size^5^. A large number of SNPs and InDels were reported in tea plant^7^, however, these markers are expensive and require special skills to assay and analyze data in any typical laboratory setup. Therefore, the identification and characterization of a large number of robust, diverse, and easy-to-assay DNA markers via polymerase chain reaction (PCR) are crucial for genetic characterization of germplasm, tagging important QTLs, and trait introgression into elite tea genotypes through marker-assisted breeding.

It is also known that the tea genome contains a high proportion of repeat sequences (70–80%), the majority of which are transposable elements (TE) and other repeat-related elements. Miniature Inverted-repeat Transposable Elements (MITEs) have the structural features of DNA transposons, with terminal inverted repeats (TIRs) flanked by small direct repeats (target site duplication, TSD) at both ends of the element. MITEs are short, typically 70 bp to 800 bp in length with an AT-rich sequence. They are inserted preferentially into intergenic, adjacent to a gene, intronic, and exonic regions, thereby playing crucial roles in gene regulation and genome evolution^8^. MITE transposition in plant genomes is known to produce a wide range of variations in plants, both at the genotypic and phenotypic levels, which can help plants to adapt to different environments. On the other hand, MITE-related sequences may encode small RNAs that regulate specific target genes at the transcriptional and post-transcriptional levels^9^. Thus, MITE-derived molecular markers are excellent candidates for gene tagging, especially when targeting genes that govern quality traits in tea. In addition to MITE sequences, intronic regions of a gene often contain a plethora of repeat sequences that contribute to the diversity of genes^10^. The difference in length of an intron between individuals on a genome-wide scale is used to create DNA markers known as Intron Length Polymorphic (ILP) markers. The importance of ILP markers is due to their co-dominant nature, neutrality, ease of assay, higher reliability, and high cross-transferability across the related species^11^. Thus, genome comparison is exploited to develop potential intron polymorphism (PIP) markers by designing primers from the flanking exon sequences of an intron that vary in length^12^. A large number of ILP markers are successfully employed in different crops, such as rice^13,14^, foxtail millet^15^, onion^16^, and carrot^17^. In the current study, we report the simultaneous identification and characterization of a large number of CsMITE and CsILP markers by comparing the two tea genomes. We also propose that these markers have practical utility in the genetic characterization of tea germplasm for different traits, genetic diversity and germplasm characterization, tagging important QTLs, and the construction of linkage maps for advanced tea breeding.

## Results

### Identification and classification of CsMITEs

The prevalence of similar structural features in CsMITEs was employed for genome-wide identification of 23,170 and 37,958 potential CsMITE candidates in both the CSA and CSS tea genomes, respectively. The TSD lengths ranging from 2 to 10 bp and TIR lengths of at least 10 nucleotides were found in the identified potential CsMITE (Supplementary Table S1). Further, from these potential candidates, 180 representative MITEs were found to be part of conserved known families, and the rest 22,990 were novel in the CSA genome, whereas 377 MITEs were found to be part of conserved known families and 37,581 were found as novel sequences in the CSS genome. These 180 and 377 CsMITEs were subsequently classified into 43 and 60 CsMITE families and superfamilies in the CSA and CSS genomes, respectively. Some of the important CsMITE superfamilies identified in the present analysis are *hAT-like, Tc1/Mariner*, *Mutator-like, PIF/Harbinger,* and *CACTA* (Supplementary Table S2**)**. The DTA Mae1 (superfamily *hAT-like*) and DTT Zem3 (superfamily *Tc1/Mariner*) families have a maximum of 27 CsMITE sequences in the CSA genome each, whereas the DTT Zem3 (superfamily *Tc1/Mariner*) family has 65 CsMITE sequences in the CSS genome (Tables 1 and 2).

**Table 1:** Classification of CsMITE families and superfamilies in the CSA tea genome.

Sr. No.	Family	Superfamily	Number of sequences
1	DTA_Mae1	hAT	27
2	DTT_Zem3	Tc1/Mariner	27
3	DTM_Phd2	Mutator	10
4	DTA_Mae3	hAT	10
5	DTM_Glm14	Mutator	9
6	DTA_Zem61	hAT	9
7	DTH_Met17	PIF/Harbinger	8
8	DTA_Met32	hAT	7
9	DTH_Zem34	PIF/Harbinger	6
10	DTA_Zem8	hAT	6
11	DTA_Viv2	hAT	6
12	DTM_Cis32	Mutator	5
13	DTA_Met30	hAT	5
14	DTM_Prp17	Mutator	4
15	DTM_Glm57	Mutator	4
16	DTM_Cac11	Mutator	4
17	DTA_Loj32	hAT	3
18	DTA_Brd27	hAT	3
19	DTT_Jac4	Tc1/Mariner	2
20	DTA_Cac3	hAT	2
21	DTT_Met26	Tc1/Mariner	1
22	DTM_Prp29	Mutator	1
23	DTM_Ors93	Mutator	1
24	DTM_Mad8	Mutator	1
25	DTM_Mad28	Mutator	1
26	DTM_Mad10	Mutator	1
27	1 DTM_Loj38	Mutator	1
28	1 DTM_Eug6	Mutator	1
29	1 DTH_Viv16	PIF/Harbinger	1
30	1 DTH_Cas3	PIF/Harbinger	1
31	1 DTA_Zem10	hAT	1
32	1 DTA_Sol2	hAT	1
33	1 DTA_Ors67	hAT	1
34	1 DTA_Met14	hAT	1
35	1 DTA_Met10	hAT	1
36	1 DTA_Loj23	hAT	1
37	1 DTA_Loj13	hAT	1
38	1 DTA_Frv18	hAT	1
39	1 DTA_Eug1	hAT	1
40	1 DTA_Cis5	hAT	1
41	SotP12		1
42	SotM34		1
43	SotM11		1

**Table 2:** Classification of CsMITE families and superfamilies in the CSS tea genome.

Sr. No.	Family	Superfamily	Number of Sequences
1	DTT_Zem3	Tc1/Mariner	65
2	DTM_Cis32	Mutator	41
3	DTA_Mae1	hAT	27
4	DTA_Zem61	hAT	27
5	DTM_Phd2	Mutator	20
6	DTA_Met32	hAT	20
7	DTH_Zem34	PIF/Harbinger	19
8	DTM_Glm14	Mutator	18
9	DTA_Mae3	hAT	15
10	DTA_Viv2	hAT	14
11	DTA_Loj32	hAT	13
12	DTA_Zem8	hAT	11
13	DTM_Prp17	Mutator	9
14	DTH_Met17	PIF/Harbinger	6
15	DTA_Brd27	hAT	4
16	SotP12		3
17	DTM_Met7	Mutator	3
18	DTM_Mae1	Mutator	3
19	DTM_Cac11	Mutator	3
20	DTH_Brr56	PIF/Harbinger	3
21	DTA_Met2	hAT	3
22	DTT_Jac4	Tc1/Mariner	2
23	DTM_Mae2	Mutator	2
24	DTM_Glm57	Mutator	2
25	DTM_Glm46	Mutator	2
26	DTM_Cil7	Mutat	2
27	DTH_Zem53	PIF/Harbinger	2
28	DTA_Viv7	hAT	2
29	DTA_Loj3	hAT	2
30	DTA_Glm3	hAT	2
31	DTA_Eug1	hAT	2
32	DTA_Brd17	hAT	2
33	Soth10		2
34	DTT_Sob1	Tc1/Mariner	1
35	DTT_Ors12	Tc1/Mariner	1
36	DTT_Met26	Tc1/Mariner	1
37	DTT_Brd32	Tc1/Mariner	1
38	DTT_Brd2	Tc1/Mariner	1
39	DTM_Prp29	Mutator	1
40	DTM_Eug2	Mutator	1
41	DTM_Cis31	Mutator	1
42	DTM_Cil10	Mutator	1
43	DTH_Jac4	PIF/Harbinger	1
44	DTH_Glm25	PIF/Harbinger	1
45	DTC_Cas1	CACTA	1
46	DTA_Zem4	hAT	1
47	DTA_Zem16	hAT	1
48	DTA_Zem10	hAT	1
49	DTA_Sol12	hAT	1
50	DTA_Sob24	hAT	1
51	DTA_Ors74	hAT	1
52	DTA_Ors60	hAT	1
53	DTA_Ors30	hAT	1
54	DTA_Met9	hAT	1
55	DTA_Met30	hAT	1
56	DTA_Loj13	hAT	1
57	DTA_Frv12	hAT	1
58	DTA_Cus1	hAT	1
59	DTA_Cac3	hAT	1
60	SotT3		1

In our analysis, around 1977 and 5466 CsMITEs were found to be located in the ‘genic’ region of CSA and CSS, respectively, whereas 1776 and 2573 were found to be located in the ‘near genic’ category in the CSA and CSS genomes, respectively. Further, a total of 18,934 and 29,144 CsMITEs were grouped in the ‘intergenic’ category in the CSA and CSS genomes, respectively. After a comparison of CsMITEs from the genic region in both CSA and CSS genomes, we found 53 and 154 unique MITEs in the CSA and CSS genomes, respectively (Supplementary Table S3). All identified CsMITEs in both the genomes were compared through a homology search to determine the common and unique CsMITEs. It was observed that 22,611 CsMITEs were shared in both the genomes, while 559 and 15,347 were found to be unique in the CSA and CSS genomes, respectively (Supplementary Table S4).

### CsMITEs as precursors of miRNA sequences

Identified CsMITEs sequences in both the genomes were used as a query to perform a homology search against downloaded 48,885 miRNAs and 522 novel *Camellia-*specific miRNAs (Supplementary Table S5). The small RNA sequences with an exact match to the CsMITE sequences were pooled as MITE-derived sequences. Among aligned CsMITEs derived miRNA sequences, we found 3964 unique miRNAs in CSA and 5198 unique miRNAs in CSS as top hits against them. Further*, Camellia*-specific novel miRNAs showed a match with 430 and 448 predicted CsMITEs from the CSA and CSS genomes, respectively.

### Identification and classification of non-redundant CsILP loci

A total of 36,951 CDS sequences from the CSA genome were searched using the online PIP marker database^12^, using *Arabidopsis thaliana* intron information as a model to find the best CsILP primer hits. The initial search designed a total of 15,087 CsILP primer pairs from 4287 unique CDSs, which matched with 3056 unique Arabidopsis CDSs. The identified CsILP primers were further filtered and removed the duplicate primer sequences, and we have assessed their unique primer binding sites on the CSA genome. This filtration process resulted in a non-redundant set of 4912 CsILP primer pairs, which have a unique primer binding site on the CSA genome and also matched to 1914 unique Arabidopsis CDS. Further, these 4912 CsILP loci mapped to 1780 scaffold sequences on the CSA genome (Supplementary Table S6). A comparison of these 4912 CsILP primer pairs to their potential primer binding sites revealed that they can bind to 4213 (85.8%) single or multiple binding sites in the CSA and CSS genomes, respectively. Out of these 4213 primers, 410 primers predicted more than one binding site in the CSS genome, whereas they were predicted to have a single binding site in the CSA genome.

### Identification of Transcription Factors (TFs) associated with CsMITEs and CsILPs

**Figure 1:**
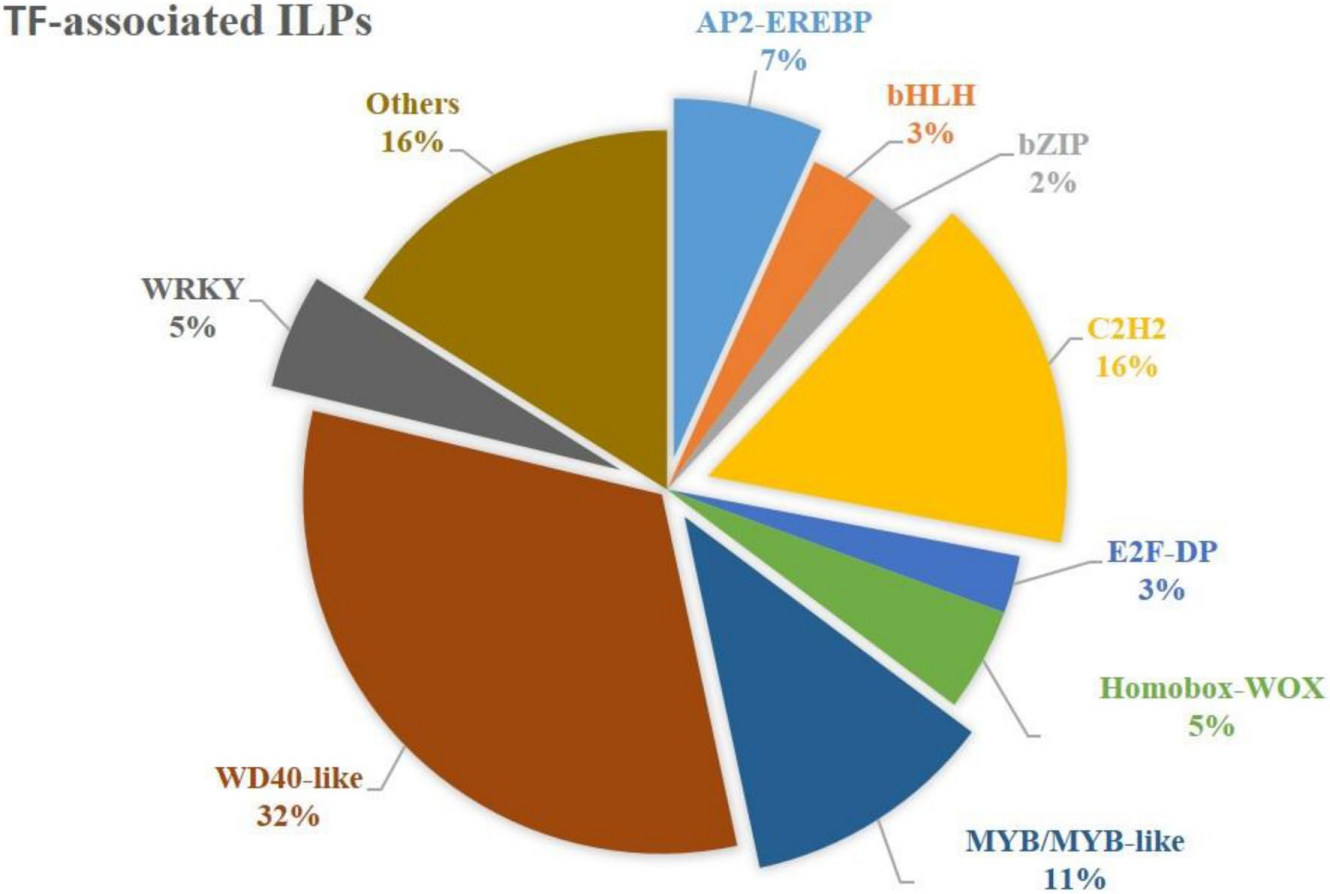
TFs associated with CsILPs in tea genomes.

The CsMITEs of both the tea genomes were searched against the Plant Transcription Factor and Transcriptional Regulator Categorization and Analysis Tool (PlantTFcat) database^18^, which harboured TFs belonging to WRKY, MYB, bZIP, bHLH, NAC, Zing finger, and AP2/ERF families. In the CSA genome, only two CsMITEs were found belonging to the bZIP and CCHC (Zn) TF families, whereas in the CSS genome, two CsMITES were found to belong to the C2H2 and HMG TF families (Tables 3A and B). A search against the PlantTFcat database produced a total of 193 hits that were relevant to TFs in the case of CsILP-containing CDS. The majority of the CsILP-containing CDS were found to be WD40-like (32%) TFs, followed by C2H2 (16%) and MYB/MYB-like (11%) TFs (Fig. 1). Other significant TFs associated with the ILP-containing CDS were AP2-EREBP (7%) and WRKY (5%), Homeobox-WOX (5%), E2F-DP (3%), bHLH (3%), bZIP (2%) and others (16%).

**Table 3:** Transcription factors identified in CsMITEs (A) CSA genome (B) CSS genome.

Family	Family_type	Sequence_Acc	Domains	Sequence_Annotation
**A**				
CCHC (Zn)	Transcription factor interactor and regulator	MITE_T_8963|xpSc0055462|26,799|27,277|ATGGAAGG|17|F973_ORF + 1	IPR001878	TSD_IN:no MITE_LEN:478 TIR_LEN:17 CANDIDATE_ID:MITE_CAND_177405
bZIP	Transcription factor	MITE_T_10766|xfSc0016954|822|1217|TA|37|F1140_ORF + 3	IPR004827	TSD_IN:yes MITE_LEN:395 TIR_LEN:37 CANDIDATE_ID:MITE_CAND_1778737
bZIP	Transcription factor	MITE_T_10767|xfSc0016954|831|1222|AT|13|F1140_ORF + 3	IPR004827	TSD_IN:yes MITE_LEN:391 TIR_LEN:13 CANDIDATE_ID:MITE_CAND_1778740
**B**				
C2H2	Transcription factor	MITE_T_27376|Scaffold120_CSS|1,242,750|1,243,302|GT|14|F2362_ORF + 1	IPR007087	TSD_IN:no MITE_LEN:552 TIR_LEN:14 CANDIDATE_ID:MITE_CAND_3842766:
HMG	Chromatin remodeling & transcriptional activation	MITE_T_27846|Scaffold257_CSS|6,734,775|6,735,302|TAA|19|F2409_ORF + 2	IPR000116	TSD_IN:no MITE_LEN:527 TIR_LEN:19 CANDIDATE_ID:MITE_CAND_3594875 COMMON_TSD:TAA:
C2H2	Transcription factor	MITE_T_28093|Scaffold6453_CSS|929,128|929,865|TA|18|F2440_ORF + 3	IPR007087	TSD_IN:yes MITE_LEN:737 TIR_LEN:18 CANDIDATE_ID:MITE_CAND_1289219:

### Gene ontology (GO) annotation of CsMITEs and CsILP loci

**Figure 2:**
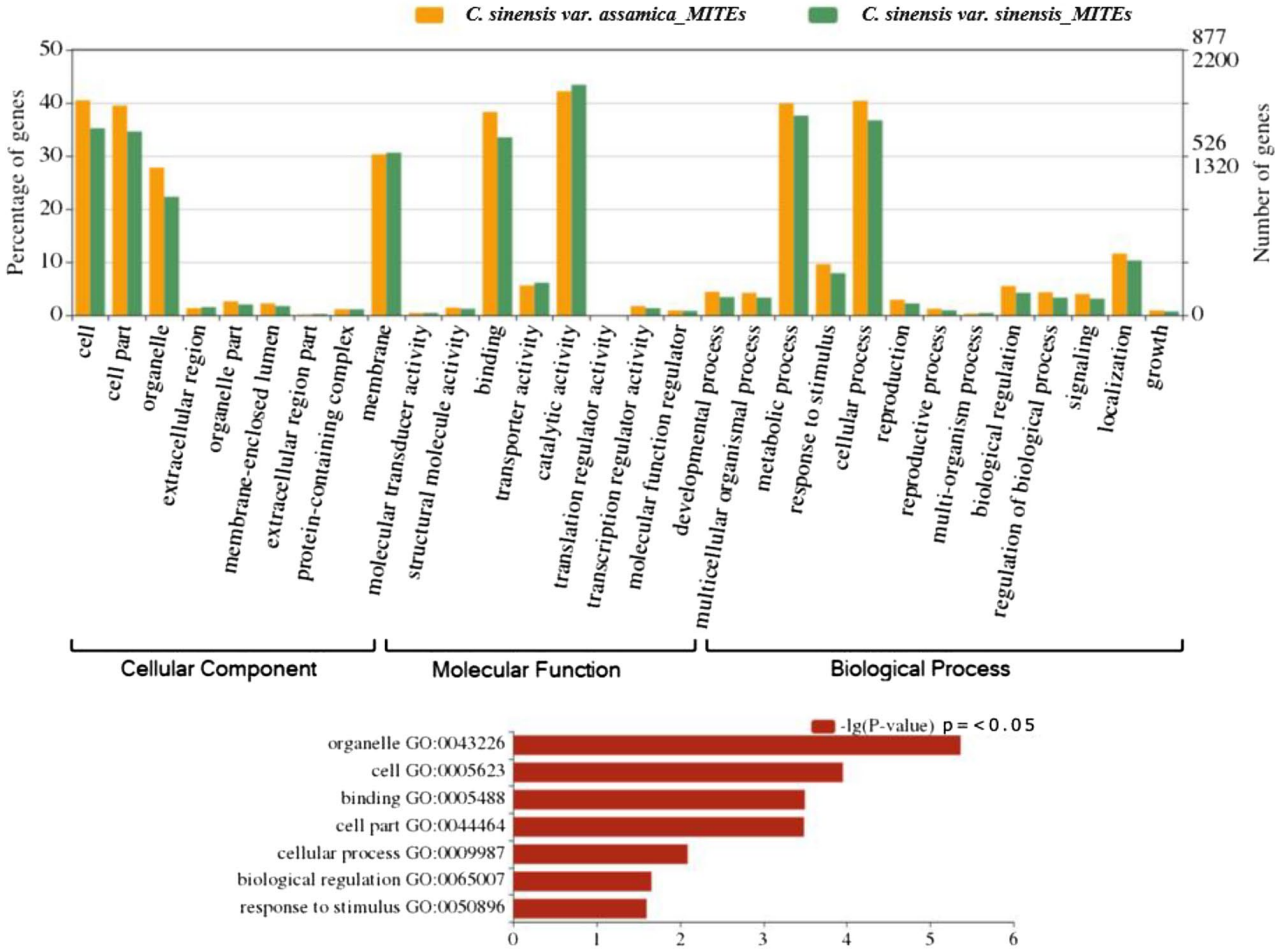
Gene ontology of CsMITEs detected in the genic region of CSA genome and CSS genomes using the online AgriGO v2.0—GO via the customized Singular Enrichment Analysis (SEA) tool against the Arabidopsis reference background annotation data (TAIR10_2017).

Functional annotation of genic CsMITEs from both the CSA and CSS genomes may help in understanding their roles in biological processes, molecular functions, and biological pathways. Therefore, GO annotation of the genic CsMITEs in both the CSA (1754 sequences) and CSS (4401 sequences) genomes was performed using the basic BLAST2GO software^19^. A total of 1328 (75.7%) and 3217 (73.1%) CsMITE sequences of the respective CSA and CSS genomes were annotated with at least one GO term associated with the cellular component (CC), molecular functions (MF), or biological process (BP). The AgriGO singular enrichment analysis (SEA)^20^ was performed against the reference database, The Arabidopsis Information Resource (TAIR) genome locus (TAIR10_2017)^21^, revealed 23 and 22 significantly enriched GO terms with FDR ≤ 0.05, respectively, for the genic CsMITEs from the CSA and CSS genomes. The blastx analysis indicated that 1624 (92.6%) and 4059 (92.2%) genic CsMITE sequences, separately from the CSA and CSS genomes, produced hits against the NCBI (National Centre for Biotechnology Information) customized plant non-redundant (nr) database. The majority of the genic CsMITEs (CSA: 448 sequences, 25.5%; CSS: 990 sequences, 22.5%) found top hits with *Vitis vinifera* sequences. According to the GO level distribution, the genic CsMITE sequences from the CSA and CSS genomes under the CC category produced the most significant sequences (*p* value ≤ 0.05) for ‘cell’ (CSA: 40.5%, CSS:35.2%) followed by the ‘cell part’ (CSA: 39.5%; CSS: 34.6%) and ‘organelle’ (CSA:27.8%, CSS:22.3%). Under the MF category of GO, the highest percent of genic CsMITEs were found significant (*p* value ≤ 0.05) for the ‘binding’ (CSA: 38.3%, CSS: 33.5%) function. In the case of the BP category, the ‘response to stimulus’ (CSA: 9.6%, CSS: 7.9%), ‘cellular process’ (CSA: 40.4%, CSS: 36.7%) and ‘biological regulation’ (CSA: 5.5%, CSS: 4.2%) were found significantly high (*p* ≤ 0.05) for the genic CsMITEs in the CSA and CSS genomes. Some of these genic CsMITEs might be related to important secondary metabolite pathways such as phenylpropanoid biosynthesis, isoflavonoid biosynthesis, anthocyanin biosynthesis, isoquinoline alkaloid biosynthesis, monoterpenoid biosynthesis, tropane, piperidine, biosynthesis of secondary metabolites, and pyridine alkaloid biosynthesis, which might serve as an important resource for marker development for tea quality breeding and genetic improvement (Fig. 2) (Supplementary Table S7). In our results, CsMITEs named MITE_T_15348 and MITE_T_12375 from the CSA genome and MITE_T_831, MITE_T_16821, MITE_T_5898, and MITE_T_29409 from the CSS genome were found to be involved in caffeine metabolism. MITE_T_8758, MITE_T_23119, MITE_T_11869, and MITE_T_14708 from the CSA genome, as well as MITE_T_27732, MITE_T_19350, MITE_T_34833, and MITE_T_18111 from the CSS genome, were discovered to be involved in the phenylpropanoid biosynthesis pathway (Supplementary Table S8).

**Figure 3:**
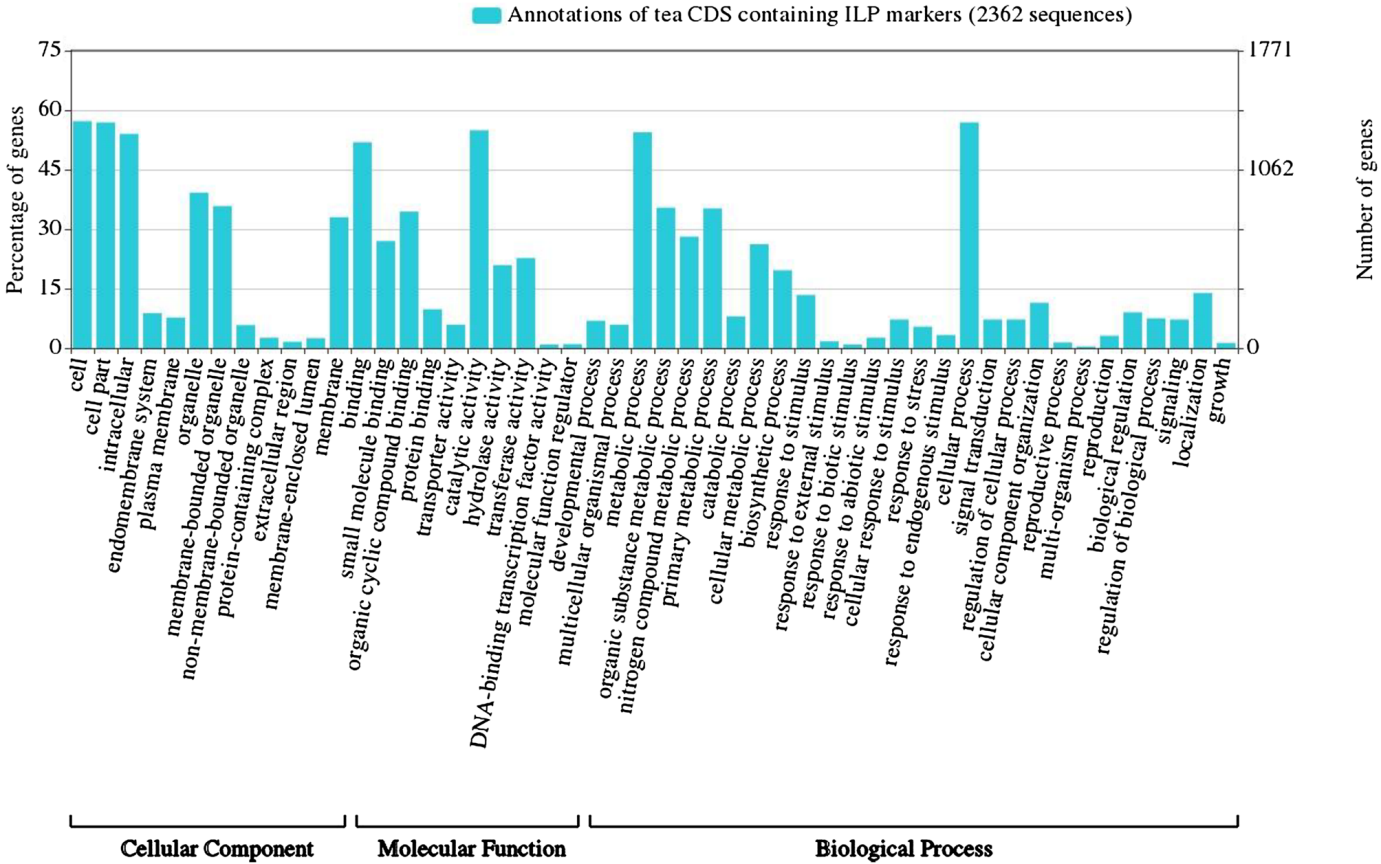
Gene ontology of CsILPs detected in the genic region of CSA genome and CSS genomes using the online AgriGO v2.0—GO via the customized Singular Enrichment Analysis (SEA) tool against the Arabidopsis reference background annotation data (TAIR10_2017).

Similarly, the GO annotation of the CsILP containing CDS showed that 2123 (89.9%) out of 2362 could be related to at least one GO term associated with the CC, MF, or BP categories. Using the AgriGO-customized SEA tool, a total of 39 significantly enriched GO terms were identified with FDR ≤ 0.05 against TAIR genome locus *(*TAIR10_2017) reference annotation data. The BLASTx analysis further indicated that the majority of the CDS sequences (2340 sequences, 99.1%) produced hits against the plant ‘nr’ database and 497 CDS sequences (21%) found top hits for *Vitis vinifera*. Among the BP category, the GO terms associated with ‘cellular process’ (34%) followed by ‘metabolic process’ (33%) were the two major GO categories exhibited in the CsILP-containing CDS sequences. Under the MF category, ‘nucleotide binding’ (31%) and ‘hydrolase activity’ (25%) were found as the major two categories. Among the GO terms associated with CC, ‘cell’ (31%) and cell part (31%) were two major GO categories determined. Besides, ‘biosynthetic processes’ (19.6%) and ‘response to stimulus’ (13.4%) were also found as two notable GO categories, which might be fascinating in tea plant genetic improvement research (Fig. 3). Some of the CsILP markers, namely CSAPIP0022, CSAPIP1511, CSAPIP1721, CSAPIP2102, CSAPIP2983, CSAPIP3308 and CSAPIP3671 were found to be involved in theanine biosynthesis related pathways (Supplementary Table S8).

### Validation of selected CsMITE and CsILP markers

**Figure 4:**
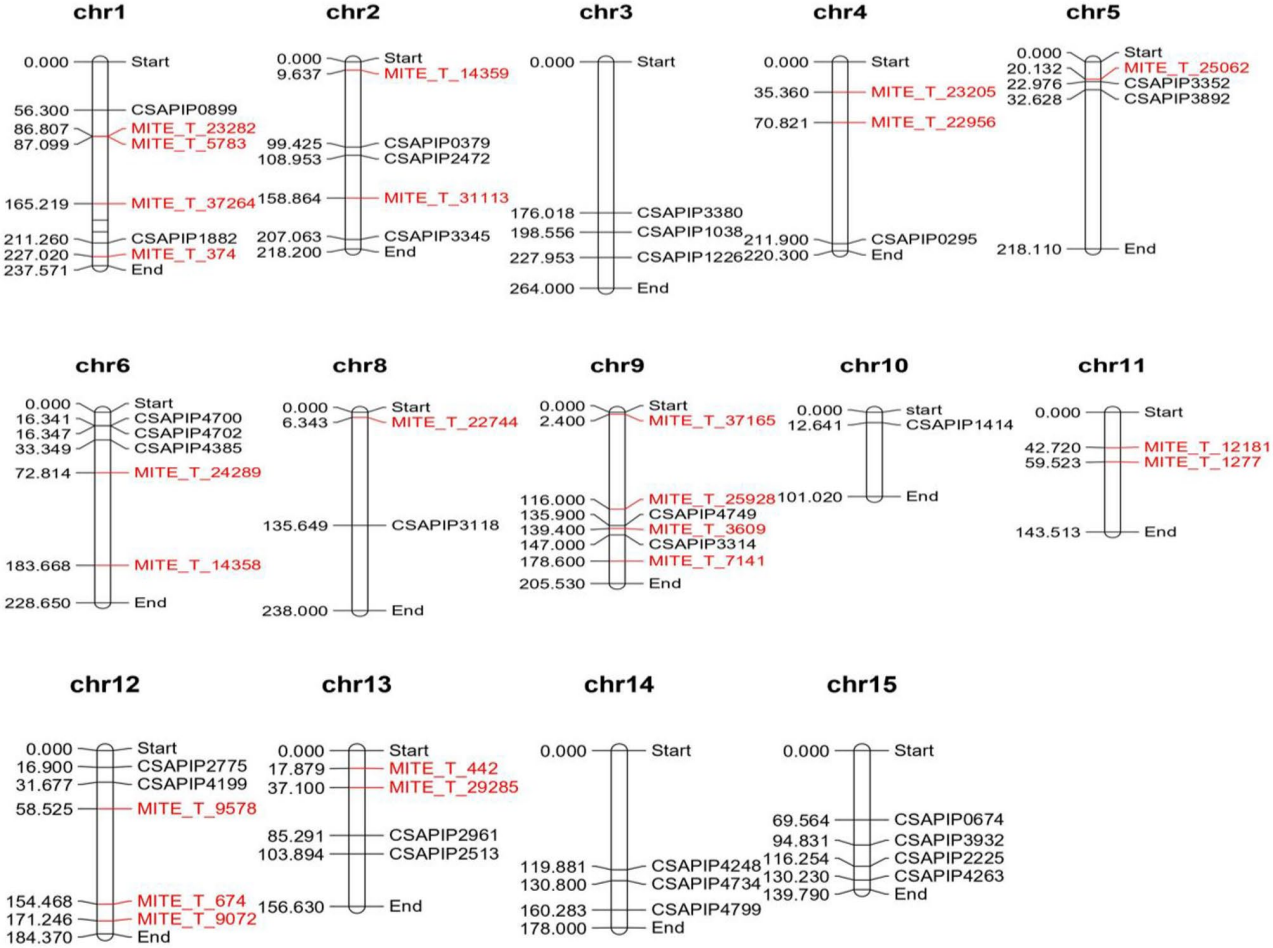
Chromosomal location of MITEs and ILP markers selected for validation generated by MapChart.

**Figure 5:**
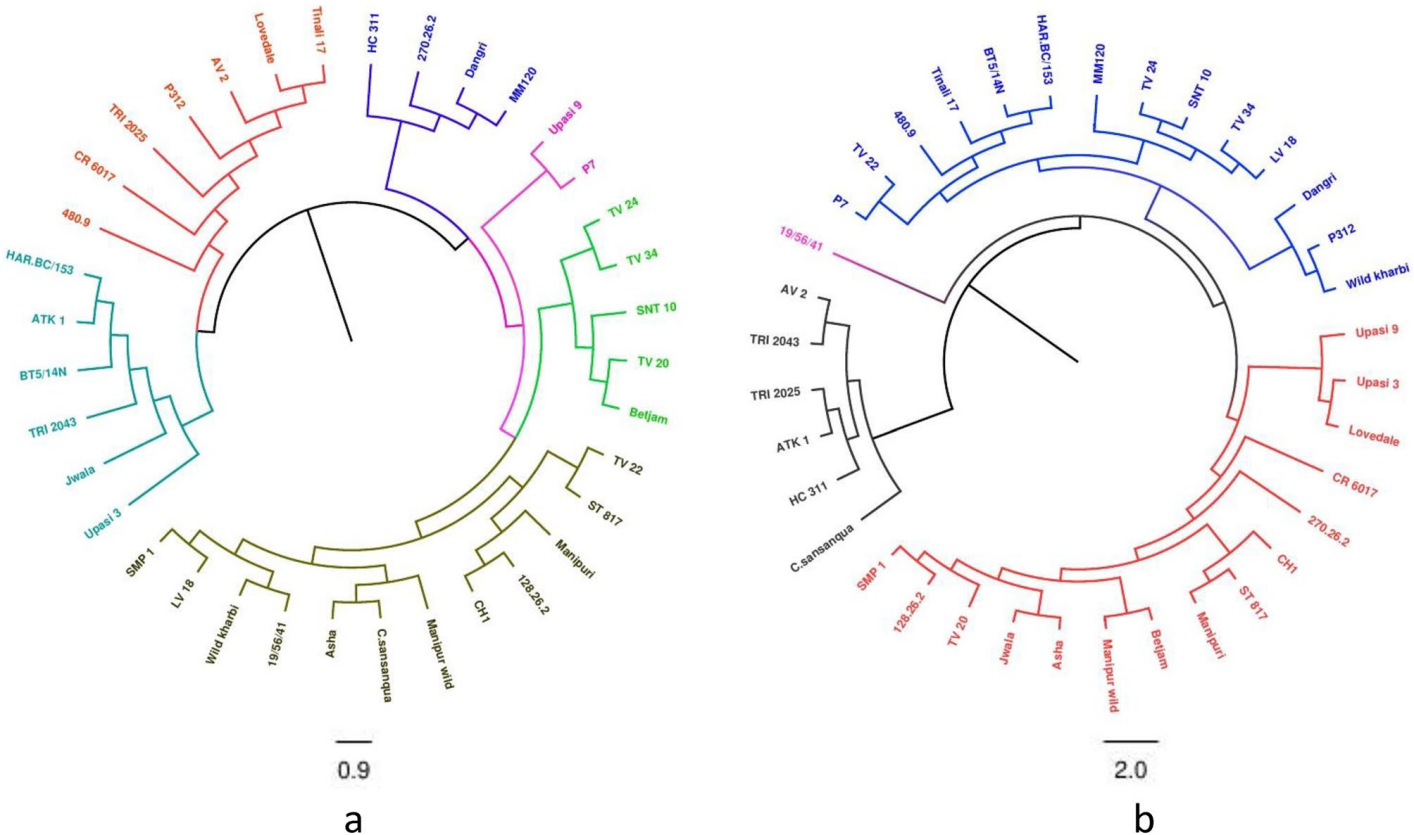
Phylogenetic tree of 36 diverse genotypes using the DARWIN 6 program with the neighbor-joining method (**a**) CsMITE markers (**b**) CsILP markers.

We randomly selected 25 CsMITE and 33 CsILP primers for validation that have single primer binding sites in the tea genomes as predicted by in silico analysis (Supplementary Table S9). These 25 MITEs and 33 ILP markers are widely distributed across all the 15 chromosomes of the tea genome (Fig. 4). Initially, nine diverse tea genotypes were chosen to screen for the polymorphism that yielded 10 CsMITEs (Supplementary Table S10) and 15 CsILP polymorphic markers (Supplementary Table S11). Later, we used 36 diverse tea genotypes for further analysis at the genotypic level. The number of alleles per locus generated by each marker varied from 1 to 4 in CsMITEs and 1–6 in CsILP-based markers. The maximum number of alleles (i.e., 4) was generated by one CsMITE named MITE_T_23247 in all 36 tea genotypes (Supplementary Fig. S1). One CsILP, namely CSAPIP1038, has generated 6 alleles after running in PAGE (Supplementary Fig. S2). A phylogenetic tree was constructed using the 10 polymorphic CsMITE markers that divided all the 36 diverse sets of genotypes into 2 different main clusters. Cluster 1 contains 2 small sub-clusters which included 6 genotypes and 7 genotypes of tea (Fig. 5a). Cluster 2 was further divided into 2 sub-clusters; one is major with 19 genotypes and another is minor with 4 genotypes.

Similarly, the 15 polymorphic CsILP marker-based phylogenetic tree clustered 36 diverse genotypes into 2 clusters, the major cluster consisting of 30 genotypes, while the minor cluster grouped 6 genotypes (Fig. 5b). The major cluster is further divided into 2 sub-clusters in which one consists of 29 genotypes, leaving a single genotype out clustered.

## Discussion

The advancement of whole-genome sequencing along with the availability of robust in silico tools can accelerate the development of low-cost, highly efficient gene-associated functional molecular markers for genotyping. The MITE-derived markers have an edge over other markers in terms of stability, and their high copy number can serve as a plentiful resource for producing genome-wide markers. Their close association with the genic regions can assist breeders to develop functional molecular markers to tag key agronomic traits.

In the present study, we took advantage of ILP and MITE polymorphic loci insertion to develop a large number of CsILP and CsMITE markers in the commercially important tea crop. The identification of MITEs is crucial as they are involved in the evolution of genomes and can significantly regulate the expression of host genes directly^12^ or through MITE-derived small RNAs^9^. We found a significant number of CsMITEs, which is about 83.464% and 78.42% located in the intergenic regions. Also, about 7.82% and 6.92% of CsMITEs were found adjacent to genic regions in the CSA and CSS genomes, respectively. Nevertheless, a considerably lower proportion of CsMITEs, i.e., 8.71% and 14.65%, were interestingly located in the genic region of the CSA and CSS genomes. A similar distribution pattern of MITEs is also reported in *Arabidopsis thaliana*^22^, *Oryza sativa* L. ssp. *japonica*^23^ and *Brassica* genomes^24^. Introns, which were previously thought to be non-coding DNA, are now known to play important roles in gene expression regulation^25^. Therefore, by harnessing the advantage of publicly available genome sequences, we identified introns in the whole genome to exploit their length polymorphism as molecular markers in plants. Among the simple PCR-based markers, ILP is gene-specific, often hypervariable, neutral to the environment, and co-dominant, which has a high transferability rate in related species^12^. Previously, genome-wide intron-derived polymorphic markers in rice^14^, foxtail millet^15^ sorghum^26^, chickpea^27^, and *Macrotyloma* spp^28^ were reported. In the present study, we developed a large number of CsILP markers (4192) from the two sequenced genomes of tea harnessing the intron derived marker development tool^12^. As a part of CDS, these CsILP markers were further characterized with GO annotations and TF association to establish their functional implications in tea germplasm characterizations and breeding.

After detection of potential CsMITEs, they were classified into superfamilies, where *Tc1/Mariner* and *hAT-*like superfamilies constitute the maximum CsMITEs in both the genomes. In previous studies, *Tc1-*like elements have also been identified in angiosperms such as *Oryza sativa*, *Brassica rapa*, *Cannabis sativa*, and *Triticum urartu*^29^. The pathway analysis with the CsMITEs from the genic region revealed their association with the important secondary metabolite biosynthetic pathways like phenylpropanoid biosynthesis, isoflavonoid biosynthesis, anthocyanin biosynthesis, and mono-terpenoid biosynthesis (Supplementary Table S7). In the current study, identified CsMITEs were found to be involved in secondary metabolite synthesis pathways were higher in the CSS genome as compared to the CSA genome. GO terms of CsMITEs from both CSA and CSS genomes showed that the highest percentage of CsMITEs fall under the ‘biological process’ category, such as response to stimulus, cellular process, and biological regulation. Similarly, based on GO term and pathway analysis of the CsILP loci in the tea genomes, numerous CsILPs could be associated with the pathways for caffeine metabolism and phenylalanine, tyrosine, and tryptophan biosynthesis. The major determinant of tea quality is the presence of bioactive compounds produced from different secondary metabolic pathways. Therefore, the genomic markers of CsILPs (e.g., CSAPIP1511-glutamine oxoglutarate aminotransferase and CSAPIP2102-glutamine synthetase for theanine synthesis) and CsMITEs (e.g., MITE_T_8010, MITE_T_33358, MITE_T_2957, MITE_T_5423, MITE_T_21237, and MITE_T_7677 for flavonoid biosynthesis, MITE_T_34833, and MITE_T_18111 for phenylpropanoid biosynthesis) related to important secondary metabolite pathways may aid in targeted breeding research in tea crop improvement (Supplementary Table S8).

In the present study, the majority of the small RNA derived-CsMITEs (i.e., 83.44% and 78.39%) were detected in the intergenic regions and only around 16.54% and 21.59% were mapped to genic and near genic regions of the CSA and CSS genomes, respectively, which was in accordance with those detected in the rice^30^. This may be due to fewer protein-coding sequences in comparison to transcriptionally active regions. The class II MITEs, during plant evolution, can act as mobile elements to shuffle TF-binding sites and modify transcriptional networks^31^. In order to find transcription factors, CsMITEs were investigated, but only two classes—bZIP and CCHC (Zn)—were found in the tea genomes. Altering bZIP gene expression patterns has been shown to influence many signalling and regulatory networks involved in a variety of physiological processes^32^, whereas CCHC (Zn) transcription factor is important for the gene expression regulation and cell cycle arrest^33^. In the present study, several CsILP loci (8.2%) were found associated with the TFs. Interestingly, the TFs or regulatory sequence-derived molecular markers were used in germplasm characterization in *Medicago sp.*^34^ and flax^35^. Therefore, identified CsMITE and CsILP markers related to TFs might serve as an important genomic resource for the characterization and tagging of agronomically important traits for tea breeding. In the current study, for the validation of CsMITE and CsILP markers, we used 36 diverse genotypes of tea, and the results were found to be very promising. We found 10 CsMITE markers to be polymorphic out of selected 25 markers and found to be involved in important pathways in the genome, e.g., MITE_T_22744 tangled with phosphate translocation and MITE_T_7141 is a TPR repeat-containing thioredoxin TTL3 involved in osmotic stress response (Supplementary Table S10). Likewise, 15 CsILP polymorphic markers were found to be involved in most of the important pathways in the genome, e.g., CsILP markers, namely CSAPIP2225, CSAPIP4702, and CSAPIP4263, were found to be involved in the biosynthesis of secondary metabolites (Supplementary Table S11). The phylogenetic tree of these 36 genotypes exposed huge variations among the 36 tea genotypes. In addition, polymorphism present in both the CsMITE and CsILP markers can be further evaluated and tested for association with the phenotypic variance of the trait, which can be successfully employed in the improvement of tea plants.

## Conclusion

The current study revealed 22,990 novel CsMITEs sequences in the CSA genome and 37,581 novel CsMITEs sequences in the CSS genome. Similarly, we found 4213 non-redundant and putative CsILP markers that were shared by both tea genomes. Annotation of the CsMITE and CsILP marker-containing sequences revealed numerous markers associated with caffeine metabolism and secondary metabolite biosynthesis pathways, such as phenylalanine, tyrosine, and tryptophan biosynthetic pathways. The validation of the markers in tea germplasm revealed polymorphism and genetic diversity, which could be useful in genotype characterization, comparative mapping, and relationships at the genomic level in the tea crop. Furthermore, the high polymorphism potential in both marker types could be exploited to associate distinct phenotypic variances in tea quality traits. This functional molecular marker resource could be used to improve tea crops and other related perennial woody plant species through marker-assisted breeding.

## Material and methods

### Identification and classification of CsMITEs

The published whole genome sequence of two tea cultivars, namely, ‘Yunkang 10’ (CSA genome) ^4^ and cultivar ‘Shuchazao’ (CSS genome) ^5^, were chosen for this study. The entire workflow is depicted in Supplementary Fig. S3. The open-source software program MITE Tracker ^36^ was used to scan both the tea genomes (CSA and CSS) to identify potential CsMITE candidates with default parameters. This program identifies putative elements first by searching for accurate inverted repeat sequences, followed by a calculation of the local composition complexity (LCC) score. After these initial steps, valid candidates are identified, followed by clustering using the VSEARCH tool^32^. These putative CsMITE candidates were then classified by aligning the sequences with the annotated MITE sequences of plant MITE databases (P-MITE)^37^using the BLASTn homology search tool ^38^ from the NCBI with an e-value cut-off of ≤ 1e^−5^.

Based on the locations within or outside the gene sequences, CsMITEs of both the CSA and CSS genomes were classified as either genic, near genic, or intergenic using BED Tools v2. 27.0^39^. The genic region is defined as a region where CsMITEs were found within a gene, near the genic region, and consists of sequences within 1000 bp sequences upstream or downstream to a gene, whereas intergenic CsMITEs were found beyond the gene but within its 1000 bp flanking regions on both sides^8^.

### Genome-wide analysis of common and unique CsMITEs

A comparative genome-wide analysis was performed to identify conserved and unique CsMITEs sequences in the CSA versus CSS tea genomes through a sequence similarity approach. The similarity search was performed using the BLASTn program with an e-value cut-off ≤ 1e^−5^. To identify common and unique sequences in both the genomes, the CSS dataset was used as a query sequence to the database of the CSA genome, which compares one or more nucleotide query sequences to a subject nucleotide sequence or a database of nucleotide sequences. The potential sequences after the concurrent analyses in the next sections were then used to design and develop markers.

### Identification of CsMITE as miRNA precursor

A total of 48,885 miRNA sequences from 271 organisms were downloaded from miRBase version 22 biological databases^40^, and 522 novel *Camellia* specific miRNAs were manually collected from research articles^41–43^. All the CsMITE elements identified earlier in both the genomes were used as a query to perform a homology search using the BLASTn program with an e-value of 1e^−5^ against these small RNA sequences. Only the predicted CsMITEs which had a perfect top match with the small RNAs were considered as CsMITE-derived small RNAs.

### Identification and development of CsILP markers

Coding sequences of the CSA genome^4^ were used to identify potential ILP loci using the online ‘Develop’ tool of the database of PIP markers^12^. The tool to predict putative CsILP loci compared the dicot model plant, *Arabidopsis thaliana* polymorphic intron sequences. Accordingly, forward and reverse primers were designed from the 100 bp flanking sequences of an intronic sequence of ≤ 400 bp. The primers designed were first checked manually to remove the duplicate primer sequences. The resulting primer sequences were then subjected to in silico PCR against the CSA and CSS whole-genome assemblies using the ‘0’ mismatch in the Primer Search tool of EMBOSS software^44^ for detection of redundant primer-binding sites. Only the primers producing single amplimers were finally selected as potential non-redundant CsILP markers.

### In silico mining of transcription factors associated with CsMITEs and CsILPs

Transcription factors in both the CsMITE and CsILP-containing sequences of the CSA and CSS genomes were identified using the online PlantTFcat tool^18^. The potential CsMITE and the CsILP-containing sequences were taken as input to the PlantTFcat database. These sequences were searched against all the transcription factor protein sequences present in this database by analysing their InterPro Scan domain patterns.

### Annotation of CsMITEs and CsILP markers

The functional annotation and the pathways of genic CsMITEs and the CsILPs-containing CDS sequences were mapped separately using the basic BLAST2GO program GO v.5.2.5^19^. In the BLAST2GO analysis, the local BLASTx tool of NCBI BLAST^+^ version 2.3.0 was run against a customized NCBI plant nr database using an e-value cut-off at 1e^−5^. The BLAST2GO interpro scan, GO mapping, and annotation tools were used with the default settings. GO-Slim analysis using the Plant slim and GO-Enzyme code mapping and KEGG was chosen for the final annotations. Finally, the GO annotated combined graph was plotted using the web gene ontology annotation plotting tool WEGO 2.0^45^ at GO level 2 data. Additionally, the GO enrichment analysis was performed using the online AgriGO v2.0^20^ via the customized Singular Enrichment Analysis (SEA) tool against the Arabidopsis reference background annotation data (TAIR10_2017)^21^.

### Validation of CsMITEs and CsILPs markers

In total, 50 CsMITE sequences, i.e., ten each of short, medium, and long conserved sequences and 20 from novel CsMITE sequences of both the genomes, were used to design primer pairs from the flanking regions. For validation, a total of 25 primers were chosen that were anticipated to have single binding sites on both tea genomes (Supplementary Table S9). Similarly, out of 4912 CsILP-based markers, a total of 33 primers with more than 200 bp were selected (Supplementary Table S9). A chromosomal map was constructed which displayed the locations of 25 MITEs and 33 ILP markers distributed across all 15 chromosomes of tea using MapChart^46^. These CsILP-based primers have only one amplimer and have significant differences in amplicon size in two different tea genomes (in silico PCR). For validation of CsMITEs, we used 36 diverse tea genotypes (Table 4) to check polymorphism using CsMITEs and CsILPs-based markers. Genomic DNA was extracted by following the standard protocol^47^. Each PCR reaction was carried out in a 25 μL total volume consisting of 25 ng of genomic DNA, 0.5 μM each of forward and reverse primer, 0.25 mM of each of the dNTPs, 0.5 U Taq DNA polymerase, and 1 × Taq buffer. The PCR amplifications were carried out in a PCR (Applied Biosystems™) with the following thermal profile: 94 °C (5 min.), 30 cycles of 94 °C (60 s), 58–63 °C (60 s), 72 °C (45 s) and a final step of 72 °C (7 min.). PCR products were separated in a 6% polyacrylamide gel, stained with ethidium bromide and viewed under the Gel Documentation System (Gel Doc XR^+^ system, BioRad, USA). The number of alleles was scored manually for each CsMITE and CsILP-based marker. The molecular weight marker of 100 bp was used to identify the molecular weight of the amplified products. We determined the various parameters of genetic diversity and a phylogenetic tree was constructed using the software program DARwin v.6.0^48^.

**Table 4:** 36 diverse genotypes of Tea used for validation of CsMITEs and CsILPs.

S. No.	Names	Features
1	CR 6017	High flavour
2	UPASI-9	Drought tolerant
3	TRI-2043	High pubescence content
4	UPASI-3	Triploid standard clone
5	Lovedale	Dwarf type
6	ATK-1	High flavour
7	TRI-2025	High flavour
8	*C. sasanqua*	Camellia species
9	AV-2	Darjeeling clone
10	P-312	Assam
11	Tinali-17	China
12	Dangri	Assam hybrid
13	BT5/14 N	China hybrid
14	480.19	Cambod china
15	MM120	Assam hybrid
16	HAR.BC/153	China Assam
17	P7	China
18	TV-22	TRA popular clone
19	19/56/41	Assam
20	HC-311	China
21	TV-24	TRA popular clone
22	TV-34	TRA popular clone
23	Wild kharbi	Wild tea
24	LV-18	Assam
25	SNT-10	High waterlogging tolerant
26	TV-20	TRA popular clone
27	Betjam	Assam
28	Asha	popular clone of kangra valley
29	Jawala	Clone
30	128.26.2	China
31	270.26.2	Cambod type
32	Manipur wild	Wild tea
33	SMP-1	Blister blight disease tolerant
34	ST-817	Highly pigmented
35	Manipuri	Assam hybrid
36	CH-1	Very small leaf

## Supplementary Information

Supplementary Fig. S1.

Supplementary Fig. S2.

Supplementary Fig. S3.

Supplementary Table S1.

Supplementary Table S2.

Supplementary Table S3.

Supplementary Table S4.

Supplementary Table S5.

Supplementary Table S6.

Supplementary Table S7.

Supplementary Table S8.

Supplementary Table S9.

Supplementary Table S10.

Supplementary Table S11.

## Data Availability

The published whole genomic sequence data of two tea cultivars, namely ‘Yunkang 10’ CSA genome^4^ and the cultivar ‘Shuchazao’ CSS genome^5^, was used as a reference in the present study. All the data generated in this study have been provided as a supplementary files along with this manuscript.

## References

[1] Mondal TK (2014). Breeding and biotechnology of tea and its wild species.

[2] Mondal T, Bhattacharya A, Ahuja P, Chand P (2001). Transgenic tea [*Camellia sinensis* (L.) O. Kuntze cv. KangraJat] plants obtained by Agrobacterium-mediated transformation of somatic embryos. Plant Cell. Rep..

[3] Mukhopadhyay M, Mondal TK, Chand PK (2016). Biotechnological advances in tea (*Camellia sinensis [L.] O. Kuntze*): A review. Plant Cell Rep..

[4] Xia EH, Zhang HB, Sheng J, Li K, Zhang QJ, Kim C, Zhang Y, Liu Y, Zhu T, Li W, Huang H (2017). The tea tree genome provides insights into tea flavor and the independent evolution of caffeine biosynthesis. Mol. Plant.

[5] Wei C, Yang H, Wang S, Zhao J, Liu C, Gao L, Sun J (2018). Draft genome sequence of *Camellia sinensis* var. sinensis provides insights into the evolution of the tea genome and tea quality. Proc. Natl. Acad. Sci. USA.

[6] Chang Y, Oh EU, Lee MS, Kim HB, Moon DG, Song KJ (2017). Construction of a genetic linkage map based on RAPD, AFLP, and SSR markers for tea plant (*Camellia sinensis*). Euphytica.

[7] Liu S, An Y, Tong W, Qin X, Samarina L, Guo R, Xia X, Wei C (2019). Characterization of genome-wide genetic variations between two varieties of the tea plant (*Camellia sinensis*) and development of InDel markers for genetic research. BMC Genom..

[8] Sampath P, Lee SC, Lee J, Izzah NK, Choi BS, Jin M, Park BS, Yang TJ (2013). Characterization of a new high copy Stowaway family MITE, BRAMI-1 in Brassica genome. BMC Plant Biol..

[9] Portnoy V, Huang V, Place RF, Li LC (2011). regulating gene expression and genome evolution RNA and transcriptional upregulation. Wiley Interdiscip. Rev. RNA.

[10] Stemmler MP, Hecht A, Kemler R (2005). E-cadherin intron 2 contains cis-regulatory elements essential for gene expression. Development.

[11] Braglia L, Manca A, Mastromauro F, Breviario D (2010). cTBP: a successful intron length polymorphism (ILP)-based genotyping method targeted to well defined experimental needs. Diversity.

[12] Yang L, Jin G, Zhao X, Zheng Y, Xu Z, Wu W (2007). PIP: a database of potential intron polymorphism markers. Bioinformatics.

[13] Wang X, Zhao X, Zhu J, Wu W (2005). Genome-wide investigation of intron length polymorphisms and their potential as molecular markers in rice (*Oryza sativa* L.). DNA Res..

[14] Badoni S, Das S, Sayal YK, Gopalakrishnan S, Singh AK, Rao AR, Agarwal P, Parida SK, Tyagi AK (2016). Genome-wide generation and use of informative intron-spanning and intron-length polymorphism markers for high-throughput genetic analysis in rice. Sci Rep..

[15] Muthamilarasan M, Venkata Suresh B, Pandey G, Kumari K, Parida SK, Prasad M (2014). Development of 5123 intron-length polymorphic markers for large-scale genotyping applications in foxtail millet. DNA Res..

[16] Jayaswall K, Sharma H, Bhandawat A, Sagar R, Yadav VK, Sharma V, Mahajan V, Roy J, Singh M (2019). Development of intron length polymorphic (ILP) markers in onion (*Allium cepa* L.), and their cross-species transferability in garlic (A. sativum L.) and wild relatives. Genet. Resour. Crop Evol..

[17] Stelmach K, Podgorni AM, Machaj G, Gizebelus D (2017). Miniature inverted repeat transposable element insertions provide a source of intron length polymorphism markers in the Carrot (*Daucus carota* L.). Front. Pl. Sc..

[18] Dai X, Sinharoy S, Udvardi M, Zhao PX (2013). PlantTFcat: An online plant transcription factor and transcriptional regulator categorization and analysis tool. BMC Bioinf..

[19] Conesa, A. & Gotz, S. Blast2GO: A comprehensive suite for functional analysis in plant genomics, *Int. J. Plant Genomics* (2008). 10.1155/2008/619832 PMC2375974 18483572

[20] Tian T, Liu Y, Yan H, You Q, Yi X, Du Z, Xu W, Su Z (2017). AgriGO v.20: A GO analysis toolkit for the agricultural community, 2017 update. Nucleic Acids Res..

[21] Cheng CY, Krishnakumar V, Chan AP, Thibaud-Nissen F, Schobel S, Town CD (2017). Araport11: A complete reannotation of the *Arabidopsis thaliana* reference genome. Plant J..

[22] Guo C, Spinelli M, Ye C, Li QQ, Liang C (2017). Genome-wide comparative analysis of miniature inverted repeat transposable elements in 19 *Arabidopsis thaliana* ecotype accessions. Sci. Rep..

[23] Oki N, Yano K, Okumoto Y, Tsukiyama T, Teraishi M, Tanisaka T (2008). A genome-wide view of miniature inverted-repeat transposable elements (MITEs) in rice *Oryza sativa* ssp. Japonica.. Genes Genet. Syst..

[24] Sampath P, Yang TJ (2014). Miniature inverted-repeat transposable elements (MITEs) as valuable genomic resources for the evolution and breeding of Brassica crops. Plant Breed. Biotech..

[25] Jo BS, Choi SS (2015). Introns: The functional benefits of introns in genomes. Genomics Inform..

[26] Jaikishan I, Rajendrakumar P, Madhusudhana R, Elangovan M, Patil JV (2015). Development and utility of PCR-based intron polymorphism markers in sorghum [*Sorghum bicolor* (L.) Moench]. J. Crop Sci. Biotechnol..

[27] Srivastava R, Bajaj D, Sayal YK, Meher PK, Upadhyaya HD, Kumar R, Tripathi S, Bharadwaj C, Rao AR, Parida SK (2016). Genome-wide development and deployment of informative intron-spanning and intron-length polymorphism markers for genomics-assisted breeding applications in chickpea. Plant Sci..

[28] Sharma V, Rana M, Katoch M, Sharma PK, Ghani M, Rana JC, Sharma TR, Chahota RK (2015). Development of SSR and ILP markers in horsegram (*Macrotyloma uniflorum*), their characterization, cross-transferability and relevance for mapping. Mol. Breed..

[29] Liu Y, Yang G (2014). Tc1-like transposable elements in plant genomes. Mob DNA..

[30] Lu C, Chen J, Zhang Y, Hu Q, Su W, Kuang H (2011). Miniature inverted–repeat transposable elements (MITEs) have been accumulated through amplification bursts and play important roles in gene expression and species diversity in *Oryza sativa*. Mol. Biol. Evol..

[31] Morata J, Marin F, Payet J, Casacuberta JM (2018). Plant lineage-specific amplification of transcription factor binding motifs by Miniature Inverted-Repeat Transposable Elements (MITEs). Genome Biol. Evol..

[32] Alves MS, Dadalto SP, Goncalves AB, De Souza GB, Barros VA, Fietto LG (2013). Plant bZIP transcription factors responsive to pathogens: A review. Int. J. Mol. Sci..

[33] Grabarczyk, P., Winkler, P., Delin, M., Sappa, P.K., Bekeschus, S., Hildebrandt, P., Przybylski, G.K., Volker, U., Hammer, E., Schmidt, C.A. The N-terminal CCHC zinc finger motif mediates homodimerization of transcription factor BCL11B. *Mol. Cell. Biol.* **38** (2018). 10.1128/MCB.00368-17 PMC5809685 29203643

[34] Liu W, Jia X, Liu Z, Zhang Z, Wang Y, Liu Z, Xie W (2015). Development and characterization of transcription factor gene-derived microsatellite (TFGM) markers in *Medicago truncatula* and their transferability in leguminous and non-leguminous species. Molecules.

[35] Saha D, Rana RS, Das S, Datta S, Mitra J, Cloutier SJ, You FM (2019). Genome-wide regulatory gene-derived SSRs reveal genetic differentiation and population structure in fiber flax genotypes. J. Appl. Genet..

[36] Crescente JM, Zavallo D, Helguera M, Vanzetti LS (2018). MITE Tracker: An accurate approach to identify miniature inverted-repeat transposable elements in large genomes. BMC Bioinf..

[37] Chen J, Hu Q, Zhang Y, Lu C, Kuang H (2014). P-MITE: A database for plant miniature inverted-repeat transposable elements. Nucleic Acids Res..

[38] Altschul SF, Gish W, Miller W, Myers EW, Lipman DJ (1990). Basic local alignment search tool. J. Mol. Biol..

[39] Quinlan AR, Hall IM (2010). BEDTools: A flexible suite of utilities for comparing genomic features. Bioinformatics.

[40] Kozomara A, Griffiths-Jones S (2013). miRBase: Annotating high confidence microRNAs using deep sequencing data. Nucleic Acids Res..

[41] Zhu QW, Luo YP (2013). Identification of miRNAs and their targets in tea (*Camellia sinensis*). J. Zhejiang Univ. Sci. B.

[42] Yin H, Fan Z, Li X, Wang J, Liu W, Wu B, Ying Z, Liu L, Liu Z, Li J (2016). Phylogenetic tree-informed microRNAome analysis uncovers conserved and lineage-specific miRNAs in Camellia during floral organ development. J. Exp. Bot..

[43] Suo A, Lan Z, Lu C, Zhao Z, Pu D, Wu X, Jiang B, Zhou N, Ding H, Zhou D, Liao P (2021). Characterizing microRNAs and their targets in different organs of *Camellia sinensis* var. Assamica. Genomics.

[44] Rice P, Longden I, Bleasby A (2000). EMBOSS: The European molecular biology open software suite. Trends Genet..

[45] Ye J, Zhang Y, Cui H, Liu J, Wu Y, Cheng Y, Xu H, Huang X, Li S, Zhou A, Zhang X (2018). WEGO 20: A web tool for analyzing and plotting GO annotations, 2018 update. Nucleic Acids Res..

[46] Voorrips RE (2002). MapChart: Software for the graphical presentation of linkage maps and QTLs. J. Hered..

[47] Mondal TK, Singh HP, Ahuja PS (2000). Isolation of genomic DNA from tea and other phenol rich plants. J. Plant. Crops..

[48] Perrier, X. & Jacquemoud-Collet, J. P. DARwin Software. http://darwin.cirad.fr/darwin (2006).

