# Supplementary material for: Genome-wide identification and development of miniature inverted-repeat transposable elements and intron length polymorphic markers in tea plant (Camellia sinensis)
Source: Sci Rep. 2022 Sep 28;12:16233. doi: 10.1038/s41598-022-20400-7 (PMC9519581; doi:10.1038/s41598-022-20400-7)
Supplement: Supplementary file 2 — Supplementary Fig. S2. [file 41598_2022_20400_MOESM2_ESM.pptx]

## Slide 1
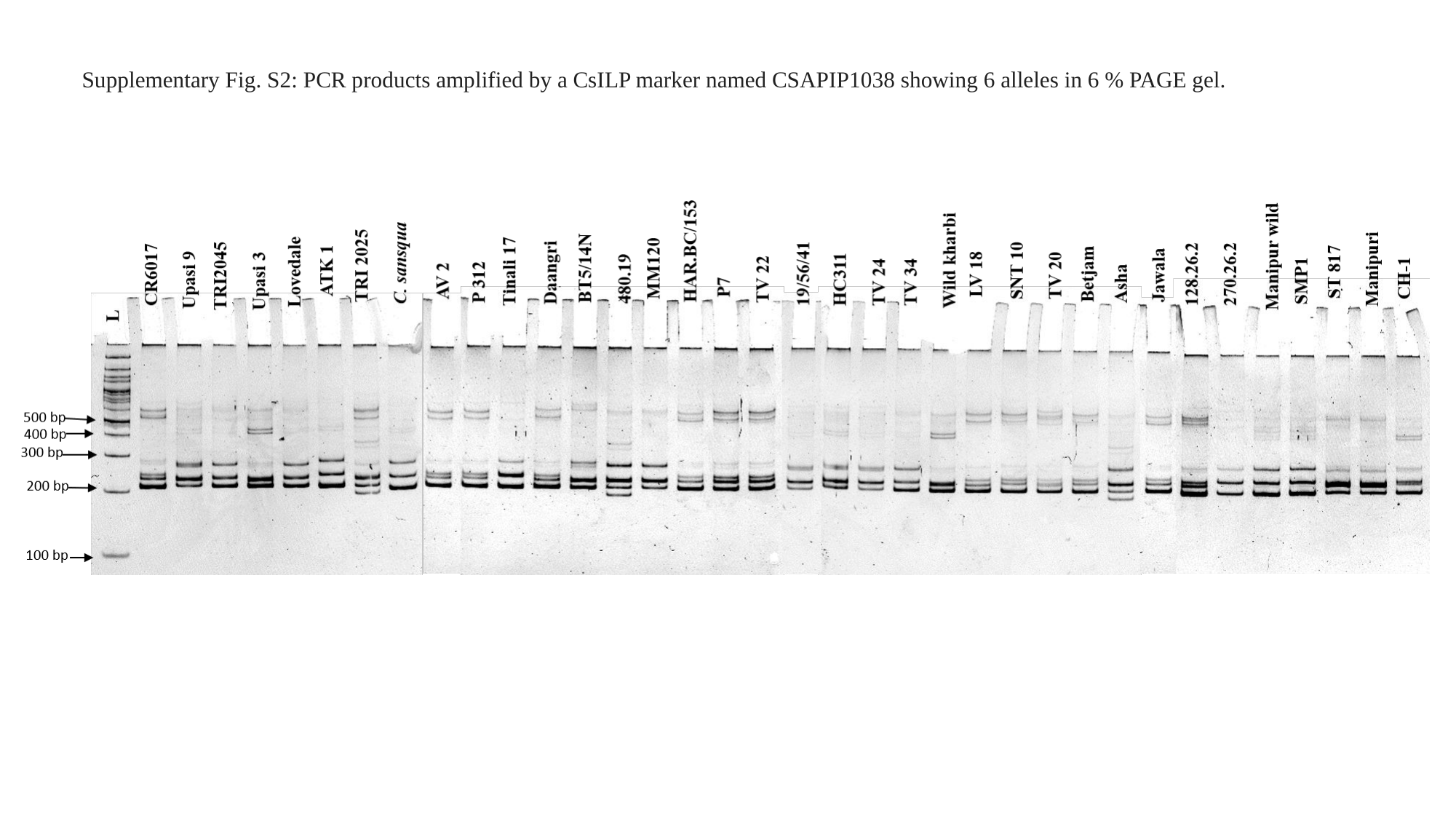

Supplementary Fig. S2: PCR products amplified by a CsILP marker named CSAPIP1038 showing 6 alleles in 6 % PAGE gel.
