# Supplementary material for: Genome-wide identification and development of miniature inverted-repeat transposable elements and intron length polymorphic markers in tea plant (Camellia sinensis)
Source: Sci Rep. 2022 Sep 28;12:16233. doi: 10.1038/s41598-022-20400-7 (PMC9519581; doi:10.1038/s41598-022-20400-7)
Supplement: Supplementary file 3 — Supplementary Fig. S3. [file 41598_2022_20400_MOESM3_ESM.rtf]

Supplementary Fig. S3: Flow diagram of the methodology used in the present study
