# Supplementary material for: Genome-wide identification and development of miniature inverted-repeat transposable elements and intron length polymorphic markers in tea plant (Camellia sinensis)
Source: Sci Rep. 2022 Sep 28;12:16233. doi: 10.1038/s41598-022-20400-7 (PMC9519581; doi:10.1038/s41598-022-20400-7)
Supplement: Supplementary file 13 — Supplementary Table S10. [file 41598_2022_20400_MOESM13_ESM.rtf]

Supplementary Table S10: MITEs polymorphic primers for validation in 36 tea genotypes


S. no.	AssaySet of CsMITEs	Start-End	Gene annotation	Size	Allele no.	
1	MITE_T_22744|Scaffold2613|806881|806993|GGCC|35|F1970_ID=TEA030417	147-503	PPT2_ARATHPhosphoenolpyruvate/phosphate translocator 2, chloroplastic 	380	4	
2	MITE_T_23205|Scaffold873|777500|777609|CA|20|F2042_ID=TEA003635	124-337	DRL27_ARATHDisease resistance protein 	235	2	
3	MITE_T_22956|Scaffold622|1032522|1032629|CA|21|F1999_ID=TEA003289	143-410	RHP16_SCHPOATP-dependent helicase rhp16 OS=Schizosaccharomyces pombe 	288	2	
4	MITE_T_23247|Scaffold556|592065|592170|CA|21|F2047_ID=TEA029567	60-427	LRK91_ARATHL-type lectin-domain containing receptor kinase IX 	390	4	
5	MITE_T_24289|Scaffold6937|1586520|1587309|GT|20|F2123_ID=TEA014264	32-1164	DLD_ARATHD-lactate dehydrogenase   	1154	2	
6	MITE_T_25928|Scaffold1318|504066|504851|CTATAG|818|F2226_ID=TEA023912	175-1028	RCF3_ARATHRNA-binding KH domain-containing protein RCF3 	850	3	
7	MITE_T_3609|Scaffold5399|286964|287221|TTTG|17|F251_ID=TEA031727	69-441	ARPC3_ARATHActin-related protein 2/3 complex subunit 3 	396	3	
8	MITE_T_7141|Scaffold1591|1847347|1847596|AT|27|F443_ID=TEA009850	29-508	TTL3_ARATHInactive TPR repeat-containing thioredoxin TTL3 	480	3	
9	MITE_T_9578|Scaffold1006|288263|288485|ATT|23|F656_ID=TEA005247	115-504	DRL27_ARATHDisease resistance protein 	411	2	
10	MITE_T_14359|Scaffold30|5095436|5095642|AGCA|38|F1074_ID=TEA009821	27-492	TA14B_ARATHTranscription initiation factor TFIID subunit 	255	3	
