# Supplementary material for: Genome-wide identification and development of miniature inverted-repeat transposable elements and intron length polymorphic markers in tea plant (Camellia sinensis)
Source: Sci Rep. 2022 Sep 28;12:16233. doi: 10.1038/s41598-022-20400-7 (PMC9519581; doi:10.1038/s41598-022-20400-7)
Supplement: Supplementary file 14 — Supplementary Table S11. [file 41598_2022_20400_MOESM14_ESM.docx]

**Supplementary Table S11: CsILP polymorphic primers for validation in 36 tea genotypes**

| **S. no.** | **ILP_Pri_IDs** | **CDS_loci** | **Gene annotation/Pathway involved** | **Start-End** | **Size** | **Allele no.** |
| --- | --- | --- | --- | --- | --- | --- |
| 1 | CSAPIP4734 | CSA001133.1 | Pyruvate metabolism, Carbon fixation in photosynthetic organisms, Carbon metabolism, Microbial metabolism in diverse environments | 1081-1574 | 200 | 2 |
| 2 | CSAPIP4248 | CSA019196.1 | RNA polymerase | 1-293 | 201 | 2 |
| 3 | CSAPIP2472 | CSA031199.1 | Synaptic vesicle cycle | 820-1315 | 204 | 2 |
| 4 | CSAPIP4364 | CSA009586.1 | Cationic antimicrobial peptide (CAMP) resistance, Necroptosis | 28-434 | 205 | 2 |
| 5 | CSAPIP3345 | CSA029082.1 | Aminoacyl-tRNA biosynthesis | 469-593 | 215 | 2 |
| 6 | CSAPIP2225 | CSA004935.1 | Citrate cycle, Carbon metabolism, Biosynthesis of secondary metabolites, Metabolic pathways, Oxidative phosphorylation | 157-1104 | 217 | 2 |
| 7 | CSAPIP1038 | CSA019856.1 | Wnt signaling pathway, Spliceosome, | 1240-1446 | 220 | 6 |
| 8 | CSAPIP4702 | CSA015773.1 | glyoxylate and dicarboxylate metabolism, Metabolic pathways, Biosynthesis of secondary metabolites | 13-191 | 239 | 5 |
| 9 | CSAPIP4799 | CSA033796.1 | cAMP signaling pathway, Cell cycle | 790-1439 | 240 | 4 |
| 10 | CSAPIP0379 | CSA012646.1 | peptide chain release factor 3, Transcription factor | 1100-1898 | 258 | 4 |
| 11 | CSAPIP3932 | CSA020068.1 | Two-component system, signal transduction | 334-560 | 270 | 2 |
| 12 | CSAPIP0295 | CSA036415.1 | Translationally controlled tumour protein | 343-503 | 295 | 2 |
| 13 | CSAPIP4263 | CSA033762.1 | Glycolysis / Gluconeogenesis, Pyruvate metabolism, Biosynthesis of secondary metabolites, Carbon metabolism, Metabolic pathways | 886-1187 | 319 | 2 |
| 14 | CSAPIP3314 | CSA012744.1 | Plant hormone signal transduction, Growth hormone synthesis, secretion and action | 458-650 | 341 | 2 |
| 15 | CSAPIP0572 | CSA004492.1 | Metabolism, TP-dependent NAD(P)H-hydrate dehydratas | 91-249 | 380 | 2 |
